# The Power of Big Data and Data Analytics for AMI Data: A Case Study

**DOI:** 10.3390/s20113289

**Published:** 2020-06-09

**Authors:** Jenniffer Sidney Guerrero-Prado, Wilfredo Alfonso-Morales, Eduardo Caicedo-Bravo, Benjamín Zayas-Pérez, Alfredo Espinosa-Reza

**Affiliations:** 1 School of Electrical and Electronics Engineering, Universidad del Valle, Cali PC 760031, Colombia; wilfredo.alfonso@correounivalle.edu.co (W.A.-M.); eduardo.caicedo@correounivalle.edu.co (E.C.-B.); 2 Integral Processes Management Department, Instituto Nacional de Electricidad y Energías Limpias, Cuernavaca PC 62490, Mexico; zayas@ineel.mx (B.Z.-P.); aer@ineel.mx (A.E.-R.)

**Keywords:** AMI, big data, clustering, data analytics, data-driven decisions, load forecasting, machine learning, smart grids

## Abstract

In recent years, there has been a transformation in the value chain of different industrial sectors, like the electricity networks, with the appearance of smart grids. Currently, the underlying knowledge in raw data coming from numerous devices can mark a significant competitive advantage for utilities. It is the case of the Advanced Metering Infrastructure (AMI). Such technology gets user consumption characteristics at levels of detail that were previously not possible. In this context, the terms big data and data analytics become relevant, which are tools that allow using large volumes of information and the generation of valuable knowledge from raw data that can support data-driven decisions for operating on the grid. This paper presents the results of the big data implementation and data analytics techniques in a case study with smart metering data from the city of London. Implemented big data and data analytic techniques to show how to understand user consumption patterns on a broader horizon, the relationships with seasonal variables identify behaviors related to specific events and atypical consumptions. This knowledge helps support decision making about improving demand response programs and, in general, the planning and operation of the Smart Grid.

## 1. Introduction

A smart grid is an electrical network that includes digital and emerging technologies to supervise and manage the operating processes for the transport of electricity from generation sources to end-users [1]. The main aim of smart grids is to optimize operational capacity as well as integrate new technologies and renewable energy sources to improve efficiency, reliability, and safety while reducing the environmental impact with economic and social benefits. Among the new applications incorporated by smart grids are emerging technologies for energy storage, smart metering integration, distribution automation for rapid failure detection, and real-time demand response [2,3].

Smart grids also promote the integration of new information and communication technologies (ICT) to achieve bidirectional communications and automated control. This combination of traditional and new digital technologies adds intelligence to the network since it increases operational capacity to acquire, communicate, process, analyze, and protect data automatically. Such intelligence allows the operator to have a better situational awareness of the network to enhance control by improving the real-time response to variations that may arise on the grid, which means a contribution towards the interoperability of the grid for the safe and reliable exchange of information [4]. However, a smart grid is technologically and economically viable only if the stakeholders involved can overcome several challenges in the areas of available energy resources evaluation, improvement of control, management, and monitoring systems. The latter includes a detailed observation of the operation and electricity demand, which implies establishing advanced metering systems that allow constant monitoring of the energy supply (utility) side and demand (client) side. In this regard, the incorporation of big data systems as a technological option offers the possibility to analyze data at nearly all stages in the energy supply process [5].

Since the smart grid incorporates significant processes like generation, transmission, and distribution of electricity, the installation for each of these processes includes a wide variety of equipment like generators, turbines, transformers, switches, current, and potential transformers, among others. The final goal of using ICTs is to measure, monitor, and even control every point in the system. It means all assets can be governed and managed by intelligent devices from generation to consumption, and is optimized based on environmental, social, institutional, or economic restrictions. Thus, the application of ICTs in the smart grid could collect large amounts of data, which also demands the use of big data and data analytics approaches [6].

Experts from McKinsey Global Institute Energy evaluated 150 use cases from several companies resulting in a full study about the impact of data analytics on them. Their reports indicate that the energy sector is one of the nine industries with the most positive potential implications of big data and data analytics. According to that study, utilities potentially have many areas of benefit around incorporating big data and data analytics [7]. For instance, work from Tu et al. suggests big data applications for wide-area situational awareness (perceiving, understanding, and projecting events in the system), state estimation, classification, and detection of events for power distribution systems [8]. According to Schuelke-Leech et al., the main aspects in which big data research for smart grids could fit are generation, transmission, distribution, billing (interfaces with the client), markets, and regulation [9]. In particular, Zhou et al. mention four sectors around big data applications: management on the generation side, microgrids and renewable energy management, collaborative operations and asset management, and demand-side management [10]. They believe that the most important focus of big data applications for smart grids are energy (savings achieved with big data), exchange (integration with other sources), and empathy (satisfy needs). They call this the “Big Data 3E.” Other applications mentioned by the authors are validation and calibration of plants, demand projection in the short term, demand response, estimation of parameters for distribution systems, and security and protection systems.

The arrival of big data applications for the smart grid brings benefits for both utilities and clients. Big data provides the opportunity to monitor, correct, and better integrate smart grid technologies, extract knowledge from data flowing through the grid, and further generate value and profits for utilities and customers [9]. It can imply notable benefits like increased grid stability and reliability, the efficient use of assets, an improved experience, and customer satisfaction. Likewise, the management of data generated from different components of the grid is fundamental for the successful implementation and operation of every process in the network [8].

One of the most critical technologies in the deployment of smart grids is the advanced metering infrastructure (AMI). This technology has allowed, in recent years, the installation of a large number of smart energy meters and other measurement terminals on the end-user side [10]. Smart meters produce data every 15 min (or less), which means that traditional databases and statistical analysis are no longer enough to extract the real value inside raw data coming from such meters. In addition to operational data, there are also other data sources to manage, such as the energy market, geographic information systems (GIS), or demographic data. This whole scenario allows identifying an increasing availability of high volumes of AMI data, the rise of advanced technologies for information analysis, and a strong need to make informed decisions to improve the planning and operation of the grid.

This work finds its purpose in the confrontation of two clear challenges in the panorama previously exposed. First, there is the use of big data techniques for the handling and processing of large volumes of raw data from smart meters. These devices generate data at time intervals that previous technologies could not offer. Second, the use of data analytics techniques to process such raw data and transform it into knowledge that adds value to the company or its customers.

The case study implemented big data and data analytics techniques with energy consumption information from 5567 London households. The households participated in the low carbon London project between 2011 and 2014. Approximately 1100 customers underwent a dynamic time of use (dToU) energy price scheme in the 2013 year. Our work consisted of integrating a big data architecture and the use of data analytics techniques for advanced metering infrastructure (AMI) data analysis. The latter stage focused on generating value for the utility or its clients through different tasks: analyzing consumer behavior, energy consumption forecasting, and identifying correlations with exogenous data that may lead to better customer characterization and better grid planning and operation.

The following section presents a compilation of several applications developed around big data and data analytics for AMI data. The subsequent chapter presents the case study implemented, describing the data sources, the big data framework, and the data analytics techniques implemented. Lastly, the visualization and access stages account for how advanced information analysis can deliver, beyond a superficial description, relevant knowledge for utilities and their clients.

## 2. Big Data and Data Analytics for AMI

One essential component of the smart grid is the advanced metering infrastructure (AMI) technology since it provides utilities with a considerable amount of new information, which was not available with previous measurement systems. Data coming from AMI Smart Meters offer valuable information that utilities can use to optimize business operations or even customer service. AMI systems also provide a pervasive communication infrastructure for constant monitoring and remote control of the grid components [11].

The extensive use of smart meters allows all the stakeholders involved in the operation of the smart grid to obtain benefits in the execution of their roles, and for the client to actively participate in the electric energy value chain, which generates new markets and possibilities for business in a smart grid [12]. These features show AMI systems as a bidirectional enabler for awareness of energy consumption in the grid. On one hand, the utility can know the customers’ consumption patterns in near real-time. On the other hand, the customer becomes an active agent who knows his consumption behavior in detail and can actively impact network management, as is the case of energy efficiency or demand response programs.

With AMI, it is possible to do continuous monitoring of customer consumption, event occurrence, and power quality, as well as open possibilities for constant interaction between users and utilities. One of the most significant consequences of this type of system is the arrival of a high volume of data that has to be processed. According to data from US Energy Information Administration, the number of AMI devices went from 49.1 million to 150.8 million between 2014 and 2017 in which their significant increase was allocated to residential and commercial buildings [13,14].

This data growing extents the acknowledge about the consumption patterns of customers improving demand response programs, specifying better tariff schemes, and monitoring other details of the network. The large volume of available information requires dedicated platforms and intensive algorithms for processing.

A study by Alahakoon D. presents an overview of the smart metering process, starting from AMI, going through advanced analytics, and then reaching all smart grid stakeholders [15]. The author states two perspectives from stakeholders’ point of view: one seeks to offer some benefits from data mining and the other aims to fulfill an established need through data analytics. The same author also presents a more comprehensive study proposing the critical elements of data analysis from the smart meters: data capture, transfer and storage, technology and algorithms, and stakeholder-related applications [16]. The work puts a particular emphasis on the challenges faced with the arrival of big data and the increase of platforms based on cloud processing such as real-time data processing, regulation of pay-per-use price models, and even security and privacy issues. Wang et al. also present a review of advanced data analytics for smart meters [17]. The paper focuses mainly on the collection of descriptive, predictive, and prescriptive analytical work for three main applications pointed out by them: load analysis, load forecasting, and load management.

The work developed around data analytics applications for data coming from smart meters generally proposes two perspectives: the processing platforms/architectures and the methods and algorithms available to process the data.

On the one hand, regarding processing platforms Shyam et al. present Apache Spark as a platform to store and run analytics for applications such as automatic demand response, pricing, and real-time data streaming [18]. Liu et al. propose a hybrid solution form smart meter data analytics, combining Spark or Hive for data processing and MADlib, which is a machine learning toolkit for in-database analysis [19]. Similarly, another study focused on the performance and efficiency of some advanced data analytics platforms [20]. The authors proposed three stages in the study. The first one is the development of a performance benchmark to evaluate different platforms. The second stage offers a solution to the problem of data availability by taking into account privacy and security aspects. Lastly, the authors implement the benchmark for evaluation purposes. The evaluation shows five advanced data analytics platforms: Matlab, MADlib, System C, Hive, and Spark Streaming. They propose an algorithm to generate large realistic datasets from a small volume of real data. The work from Daki et al., also presents different concepts on data management in smart grids [21]. In their review, the authors propose an architecture for customer advanced data analytics and show various stages for data processing like data sources, integration, storage, analytics, and visualization. Stoyanov et al. developed a study to capture and consult data from Hadoop (Hive) from smart meters. The authors highlight that a centralized model is better if the volume of data is not considered high, i.e., the limits of modern hard disks [22].

On the other hand, several authors have focused on advanced data analytics approaches for process smart metering data. Jha et al. show several advanced data analytics developed on AMI data implemented in the Puducherry Smart Grid Pilot project. In that project, they make data validation, identify meter tampering and missing information, perform energy audit and accounting, and identify peak demand and consumer profiles [23]. Work from Yu et al. shows an analysis of AMI data using fuzzy systems in the Tatung University campus. They use a combination of Cascading Style Sheets and Google chart API to support enhanced reading and real-time visualization. The result of this work is the integration of data from smart meters into a web platform for consistent visualization and the presentation of basic statistics and measurements [24]. Although many of these analyses have been done in time-domain mainly, other authors use a frequency domain methodology to characterize and analyze load profiles [25].

Regarding load profile analysis, the work by Hayn et al. performs a characterization of the consumption profiles of the clients and carry out socio-demographic studies to evaluate the influence of specific technologies and appliances on their consumption patterns [26]. Ramos et al. developed a framework to characterize medium voltage (MV) users using knowledge discovering from databases (KDD) and identifying the load profiles. The proposed methodology includes pre-processing, clustering algorithms, selection, segmentation, and classification [27]. Work from Kojury-Naftchali, is focused on the self-organizing map (SOM) to obtain the characterization of the customer’s electricity consumption behavior [28]. Besides the evaluation of customer consumption patterns, some works have increased the level of detail to reach load disaggregation to identify individual uses of some domestic appliances and evaluate their electric consumption footprint. For example, the non-intrusive load monitoring, implementing hidden Markov models, and deep learning or deep sparse coding algorithms [29,30,31].

One of the most studied applications of AMI systems is load forecasting. Forecasting is a type of regression aimed to predict the future value of a variable given its past values. Such autoregressive models may or may not consider additional or exogenous variables that share the same time-series [32].

The dynamic electricity market depends on the adequate load forecasting for appropriate demand-side management and planning as well as advanced analysis of external data that influence the behavior of customers and the electricity market. Such actions guarantee efficiency and savings for both utilities and customers.

There are several reviews of different linear and nonlinear models for forecasting tasks [33,34]. Hayes et al. suggest that nonlinear models have a better performance against linear regressors for predicting this type of variable like the Nonlinear AutoRegressive eXogenous model (NARX) [35]. Authors in the literature use different approaches for energy consumption forecasting. Most of them use machine learning algorithms. Among the most common algorithms to perform load forecasting, we uncovered the random forest estimation [36,37], autoregressive models, and neural networks [38]. Some works use feature selection combined with wavelet transform or differential evolution algorithms for short-term load forecasting [39,40]. Ali et al. use singular value decomposition (SVD) to perform short term load forecasting [41]. Aman et al. also use machine learning methods to forecast energy consumption patterns in a university campus microgrid, and mention possible applications for energy consumption planning and conservation [42,43]. Recent work from Taieb et al. present a hierarchical probabilistic approach for electricity forecasting using the *MinT* approach [44].

Other authors have included external data sources for their works. For example, Liu et al. study load forecasting by implementing a Map/Reduce framework. The authors cluster geographical data according to weather conditions [45]. The work from Cui et al. reviews the concept of prosumers (simultaneous producer and customer) and the impact of social media on their generation/consumption patterns [46]. Chen et al. also study the short-term load forecasting using deep residual networks, which, when using two public datasets, showed that it is more accurate and robust compared to other state-of-the-art forecasting models [47].

Another crucial aspect of the deployment of AMI is the possibility of active participation of customers in smart grids. For example, Kwac et al. present a methodology for demand response targeting by formulating optimization techniques to solve a stochastic knapsack problem (SKP) based on high-resolution data collection [48]. Mogles et al. analyzed the effect of personalized messages through in-home displays about consumption patterns. This work shows how, with adequate customer involvement, energy savings went up to 22%, and the energy literacy went from 0.52 to 1.28 on a scale from zero to four [49]. Work from Tascikaraoglu, A. presents a compilation of works focused on AMI data-driven demand response, which highlights the use of Artificial Neural Networks (ANN), online clustering, and distributed data analysis [4].

AMI data analysis applications do not only focus on identifying demand profiles, customer classification, or forecasting tasks. For example, Gómez Lopez et al. make an overview of AMI data potential applications for power distribution loss reduction [50]. Work from Botev et al. presents a data-driven model to identify possible sources of non-technical losses from AMI data. Their method is based on spectral analysis of periodic patterns, using features in the frequency domain. They highlight the model’s ability to perform online analysis [51]. One of the most recent surveys on the subject presents a review of machine learning techniques to detect energy theft using smart metering data. The work suggests that these techniques can be simple (supervised or unsupervised learning) or hybrid. The later technique combines any of the above with more sophisticated methods like extreme learning machines, genetic support vectorial machines, and Boolean rules fuzzy logic SVM. The authors point out how challenges in energy theft detection have not adequately been addressed yet. Such challenges can be data imbalance (normal samples in the same range), Big Data’s 3V (volume, velocity, and variety), feature description and selection, and non-malicious factors (change of residents or appliances, or seasonality) [52].

The privacy of customer data is also a significant challenge faced by developments around AMI data, which are consistently available with precise details about their consumption habits. In this sense, Foreman et al. present a methodology to anonymize customer data with smart meters installed on their properties, while preserving the billing services and automatic connection to the centralized system of a utility [53].

Considering emerging technologies, Bereş et al. present a study of several tools based on cloud computing for data analytics. The work mentions the benefits of considering aspects like security, availability, and reliability. Moreover, they show the possibility to process data in real-time safely [54]. Yan et al. also present a fog computing model to process AMI data. This approach mentions several challenges like expansion flexibility, efficiency, reliability, and high costs associated with cloud computing for AMI applications [55].

Next, Table 1 presents an overview of some works related to AMI data applications mentioned so far.

Although Table 1 presents several applications based on AMI data, it is necessary to have a development framework with an adequate methodology to transform raw AMI data into usable knowledge according to the required application. The National Institute of Standards and Technology (NIST) proposed a reference architecture framework for the development of big data projects [56]. Considering the surrounding concepts of big data, not only for AMI projects but for any application, we used the NIST reference architecture framework.

### Big Data Reference Architecture

**Figure 1:**
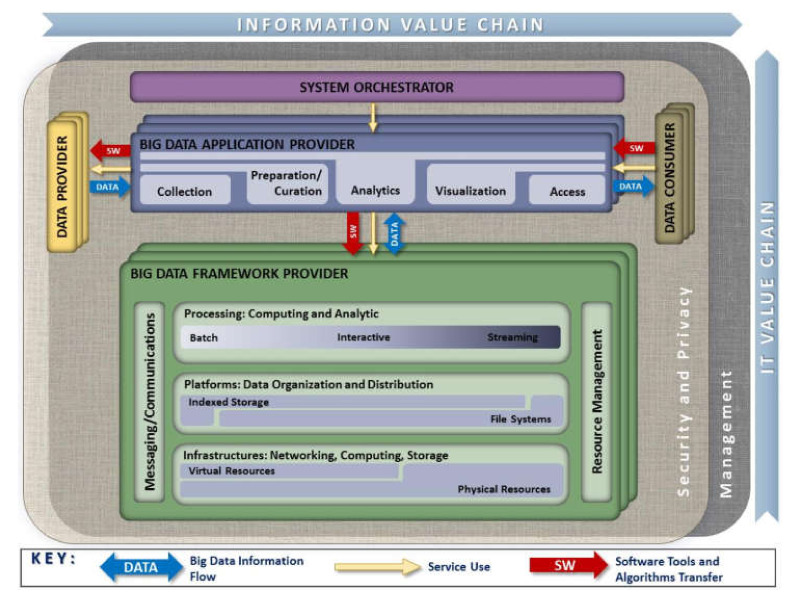
The National Institute of Standards and Technology (NIST) big data reference architecture. Source: Reference [58].

Reference architectures generally serve as a template for developing solutions in an orderly manner in a specific field and may be used for comparison and alignment purposes. The architecture proposed by NIST brings together common elements found in different documented case studies around the world. The reference architecture presented in Figure 1 also includes general considerations on big data, its implications, and requirements [57].

Five primary roles compose the reference architecture.

1. System Orchestrator: it defines and integrates the required data application activities into an operational vertical system. It provides the overarching requirements about business ownership, governance, data science, and system architecture.
2. Data Provider: it introduces new data or information sources into the big data system, either online or offline. It is also responsible for data persistence (hosting), data scrubbing (remove PII – personally identifiable information), metadata (for history and repurposing), policy for others’ access to data, and query without transferring (sometimes).
3. Big Data Framework Provider: supplies a computing infrastructure while protecting the privacy and integrity of data. Some resources or services used by the big data application provider are infrastructure framework (networking, computing, storage, environmental), data platform (physical storage, file systems, logical storage), and processing (software support for applications).
4. Big Data Application Provider: it executes a life cycle to meet security and privacy requirements. It also develops system orchestrator-defined requirements, mechanisms to capture data, preparation, analytics (discovery for finding value in big volume datasets), visualization (exploratory, explicatory, or explanatory), and access to the results of the data system.
5. Data Consumer: includes end-users or other systems that use the results of the big data application provider: search and retrieve, download, analyze locally, and reporting and visualization.

The data consumer role is in charge of understanding the results. For this, the tasks of visualization and access to the data developed by the application provider are of great importance. On the one hand, visualization allows the results of the analysis carried out to be communicated to an audience to facilitate interpretation and understanding [58]. On the other hand, the access stage allows the information to be delivered to the data consumer efficiently according to their activity or job [59,60].

In a utility, there can be different types of data consumers including developer engineers, operational coordinators, decision-makers, and others. Depending on their role in the operation of the utility, each one requires different access to information aggregated or disaggregated at a certain level. An operation coordinator may require detailed operational data about events or clients, while an executive officer may require more comprehensive results on the performance and economy of the utility. Visualization and access to the correct type of information facilitate the communication of results in the way that each individual requires [61].

One of the chapters of the framework presented by NITS documents 51 use cases in industries referring to big data and data analytics applications [62]. From all 51 documented use cases, the only one related to smart grid and AMI data is called “Machine Learning for Demand Forecasting in Smart Grids.” This case studied machine learning methods for energy forecasting consumption patterns in the USC campus microgrid, which could be useful for energy planning and conservation [42,43]. The case study presented by NIST shows an increasing need in the energy sector for implementing applications related to data processing and analysis to improve the operation of the smart grid.

So far, we presented an overview showing the importance of AMI deployment within smart grids, given the variety of applications that can potentially generate value for a utility and its customers. A review of some developments focused on the processing of a large volume of AMI data generated by smart meters using big data and data analytics techniques. The model formulated by NIST, described above, stands out as a development framework for big data applications.

As motivators for this development, it is necessary to consider the importance of the role of AMI in the smart grid, the availability of a high volume of AMI data, and the growing need for developments focused on analysing this raw data. In this way, the main objective of this work is to implement an exploratory study case that allows us to demonstrate the potential of big data and data analytics techniques applied to AMI data processing.

According to the roles depicted by the architecture, for the case described in this paper, there is a big data framework provider in charge of implementing the information value chain. In addition, a data provider includes mainly AMI data coming from smart meters and some exogenous data. The objective is to develop an application acting as a big data application provider that covers the entire process, from collection to final access so that utilities can use that data as the data consumer.

The next section describes the case study in detail. The case study presented is exploratory, i.e., the aim was to explore the benefits of different techniques (or at least some) of data analytics techniques applied in an AMI dataset to extract knowledge from raw data. This exploration includes implementing machine learning algorithms for descriptive and predictive analysis. However, a utility can require implementing more sophisticated analytical techniques for a very particular purpose, e.g., the identification of consumption patterns of a customer sector, the financial evaluation of one of its demand response programs, or the identification of losses at different points in the grid.

## 3. Case Study: Big Data and Data Analytics for Smart Meters

An exploratory case study used smart metering data published by the London Datastore from the Mayor’s Office of London. Data was collected by UK Power Networks as part of the Low Carbon London Program to investigate the impact of different low carbon technologies in the London electricity distribution network [63]. For this purpose, UK Power Networks installed around 5500 smart meters in the city of London. The meters recorded energy consumption at 30-min intervals between 2011 and 2014. During 2013, a group of 1100 households participated in a Demand Response Program using a Dynamic Time of Use tariff (ToU Group). This group had a variable electricity tariff throughout the day: high (67.20 GBX/kWh), low (3.99 GBX/kWh), or normal (11.76 GBX/kWh), while other households had a uniform rate of 14.228 GBX/kWh (Std Group).

### 3.1. Data Sources

Three sources of data were collected (meter data, weather data, and holiday data). The gathered data consisted of two parts: meter records and customers’ tariff. The first and most significant part contains around 160 million records from 2011 to 2013. The size of the raw dataset was near 11 GB, which includes the following fields.

- Unique household identifier,
- Tariff program of each household (Standard or Dynamic Time of Use),
- Energy consumption (kWh per half hour) of each household,
- Date and time,
- CACI Acorn group, and
- CACI Acorn category.

Acorn is a geo-demographic segmentation of the UK’s population. This classification segments households into six categories, 18 groups, and 62 types considering social factors and population behavior [64]. Acorn classification provides a general understanding of the attributes of a neighborhood by classifying postcodes into a category, group, or type. Acorn categories are affluent achievers, rising prosperity, comfortable communities, financially stretched, urban adversity, and non-categorized customers.

The second part contains the detail of the tariff applied to ToU customers throughout each day of 2013. More information is available at the London Datastore Website [65].

External datasets from weather and holidays allowed us to explore the correlation between energy consumption and exogenous variables.

- Weather data. It came from two datasets for climatic variables like temperature, humidity, pressure, visibility, sunset, and sundown, among others. The first dataset, with daily granularity, includes 30 climatic variables. The second dataset has 1-h granularity, detailing 10 weather variables. Both datasets, collected from the Dark Sky Company API, included data between the years 2011 to 2014 [66].
- Holiday’s data. A list of the official UK holidays from 2011 to 2014, was collected from UK Government Digital Service [67].

### 3.2. Big Data Framework

**Figure 2:**
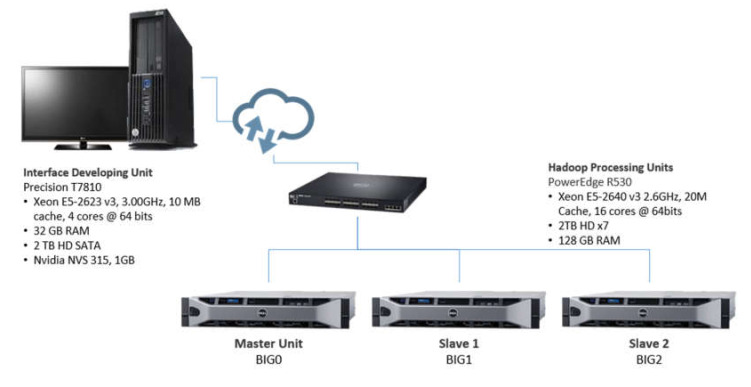
Data framework allocated for the case study.

The Big Data Laboratory of the National Institute of Electricity and Clean Energies (INEEL) played the role of big data framework provider, depicted in Figure 2.

The Apache Hadoop processing cluster consists of one master unit and two slaves. Each unit has Xeon E5-2640 processors with 16 cores, 2TB × 7 hard disks, and 128 GB RAM. All of them run Linux OS. The cluster also has big data tools such as:

- Apache HBase, the Hadoop open-source, non-relational, NoSQL, and distributed database [68].
- Apache Spark, an analytics engine for large-scale data processing [69].
- Apache Zeppelin, a web-based notebook environment that enables interactive data analytics to run different programming languages [70].

Furthermore, a workstation was networked to the processing units to provide an interface to access the master and slave nodes in the cluster.

## 4. Methods for the Big Data Analytics Application Development

The application implementation process consists of four main stages: data collection, data preparation, analysis, and visualization. We present the following.

### 4.1. Data Collection

The data collection stage implemented a Zeppelin notebook, running a PySpark interpreter to store all data collected into Spark data frames. A Spark data frame is a distributed set of data organized into columns, similar to a relational table. These data frames allow scalability of computation in processing clusters and integration with all big data tooling and infrastructure via Spark [71].

The raw data available in comma-separated values (CSV) files were imported into the processing cluster in eight data frames, as presented in Table 2.

### 4.2. Data Preparation

Data preparation is also called the ETL stage from big data lifecycle: extraction (by collecting data), transformation (by curating and preparing data), and loading (by saving data warehouse into HBase). The ETL process begins with raw data, which is the basis for building a data warehouse. The construction of the warehouse included two processes: filtering and data imputation. Filtering to discard incomplete records that cannot be estimated or that are not suitable to be considered in a specific metric (e.g., the total month consumption value cannot be calculated with incomplete records for several days in a month). Data imputation to complete missing data, when possible (e.g., a missing energy value between two time stamps for a meter, when the immediately preceding and next time instants are available).

In addition to data filtering and data imputation, it was also necessary to establish correlations and groupings between the different data sources or analyzing data with different granularity. Therefore, a data warehouse included different data frames, according to their type, granularity, or the kind of analysis to be performed.

Since our study was exploratory, we built a data warehouse with different time granularities in addition to the original raw data time-base (30 min): half-hourly, hourly, daily, weekly, and monthly. We also created data frames with information corresponding to the ACORN groups and the individual characteristics of the clients.

The initial database contained around 167 million records distributed in 8 data frames. The consolidated data warehouse stored about 676 million records distributed across 29 Spark data frames of different combinations of time granularities and grouping criteria.

This redundancy of data is then a consequence of the use of big data tools and facilitates data analytics. Therefore, it is possible to deal with complete datasets instead of a sample of data, as is done in some statistical approaches.

Although Spark data frames lie in memory, it is possible to assign some persistence of data frames on the hard disk. Nevertheless, the most appropriate way, especially if persistent access to the data is required, is to save them into the Hadoop File System (HDFS) permanently. For this purpose, the entire data warehouse was migrated to HBase using Apache Phoenix [72].

### 4.3. Data Analytics

Once we adequately transformed and stored the necessary data with different granularities, an initial descriptive analysis was necessary before using different algorithms to perform predictive analysis. We performed a descriptive visual analysis by connecting the data stored in the processing cluster to Tableau visualization tools [73].

#### 4.3.1. Descriptive Analytics

For this task, we depicted a graphic description of data with Tableau to understand the usage of energy by different user categories and time granularities.

**Figure 3:**
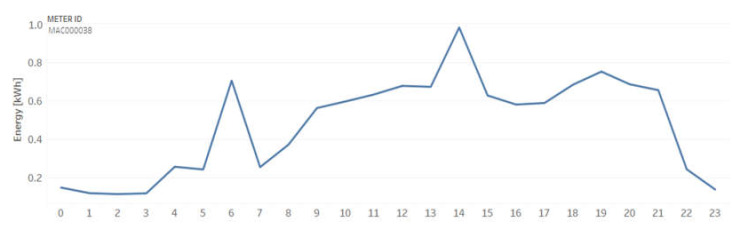
Example of a household energy consumption with a standard tariff.

For example, Figure 3 shows the energy consumption per hour of a household with standard tariff (Std) on 13 April 2013.

The case study considered five Acorn categories: affluent achievers, rising prosperity, comfortable communities, financially stretched, and urban adversity. However, rising prosperity, comfortable communities, and financially stretched groups presented reasonably similar trends. Therefore, for this study, these categories were grouped as a comfortable category. The new classification, referred in this paper as grouped categories, correspond to:

- Affluent, for all households classified as acorn affluent achievers
- Comfortable, grouping the households from rising prosperity, comfortable communities, and financially stretched categories
- Adversity for households belonging to the urban adversity acorn category.

**Figure 4:**
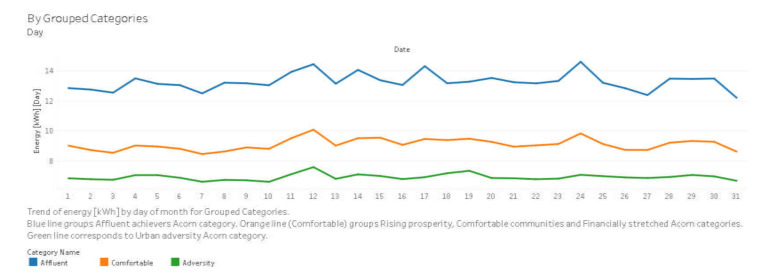
Average energy consumption for each day of August 2013 for each grouped category.

Figure 4 presents an example graph with the average energy consumption for each day of August 2013 for each grouped category.

We analyzed consumption patterns for each grouped category at different time horizons: hourly, daily, and monthly. We also made a differentiation between consumption on workdays and holidays. For each category and time horizon, we estimated average, maximum, and minimum consumption and compared users with Std and ToU tariffs.

In addition to the analysis of the consumption patterns of users and categories, we included information about exogenous variables such as weather and seasons. We analyzed the effect of variables such as temperature, daylight hours, and seasons of the year on the consumption habits of each grouped category.

Section 5 presents the results and some interesting findings from this stage of descriptive analysis. This first stage of descriptive analysis allowed us to identify different characteristics of electricity consumption in households and their categories and some relationships with other variables such as temperature and light hours. However, the power of data analytics extends its scope from the descriptive to a predictive approach, as is the case of machine learning tasks for clustering and forecasting.

#### 4.3.2. Predictive Analytics

The main goal of predictive analytics is to use current and historical information to find future patterns or characteristics not explicit in the available information [74]. In this work, we implemented two main tasks of predictive analytics: clustering and forecasting.

##### Clustering

Clustering is a machine learning task aimed to group sets of objects with similar characteristics [32]. In this case study, the households were already grouped into acorn categories and then gathered into three global categories (affluent, comfortable, and adversity), called grouped categories. Nevertheless, such geo-demographic classification segments users according to characteristics like household income and size, wealth, or social grade, but does not consider the electricity consumption patterns of each household [64]. We propose the implementation of a clustering algorithm to assign three new categories to the data: high, medium, and low, considering only electricity consumption patterns of customers, leaving aside their geo-demographic characteristics. This new categorization of users is not intended to be better or to exclude the existing segmentation, but is rather complementary.

Therefore, we now have users segmented considering two different criteria. On one hand, the criteria includes their geo-demographic characteristics and, on the other hand, the criteria involves their consumption patterns. For example, users from the adversity category (geo-demographic based) intuitively should match those users assigned by our clustering algorithm to the low consumption category (energy consumption-based). However, this expectation is not mandatory, e.g., adversity users with high energy consumptions. The possibility of having two user segmentation criteria that may or may not coincide is useful when identifying possible atypical behaviors. The evidence of possible atypical behaviors might be one of the advantages of having complementary grouping criteria.

We used K-means as a clustering algorithm. K-means proposes that a set of objects has as many centroids as groups/categories require. Each object is assigned to the group with the closest centroid to the object’s coordinates. Next, Equation (1) describes the objective function of the K-means algorithm.

(1) $$J=\overset{k}{\underset{j=1}{\sum }}\overset{n}{\underset{i=1}{\sum }}\underset{\mu_{j}\in C}{\min}\left(x_{i}-\mu_{j}{}^{2}\right),$$

where k is the number of clusters, n is the number of samples x, and $\mu_{j}$ is the centroid of each cluster.

The selected value of k=3 corresponds to the expectation of identifying three household segments, which is similar to the three grouped categories presented.

There were two possible options considered in the algorithm implementation: the Spark MLlib library [75] and the Python Scikit-learn library [76]. While the first one requires a distributed processing environment, the second is better for applications that, by their nature, do not necessarily require distributed computing. The Scikit-learn library was more appropriate for this case study, considering that the amount of elements to be grouped is small (near 5500 households). We do not need to make use of distributed computing resources, at least, for the clustering task of this specific case study. The MLlib library is more appropriate for a more significant amount of records. Moreover, it was used TSlearn, an extension of Scikit-learn, specialized in the treatment of time series data [77].

The main input arguments for the k-means clustering algorithm in TSlearn are the number of clusters (k=3) and the data set that will be grouped (5509 timelines with one for each household). These timelines have 24 measurements including one for each hour of the day, which forms a consumption pattern of each client over a day.

**Figure 5:**
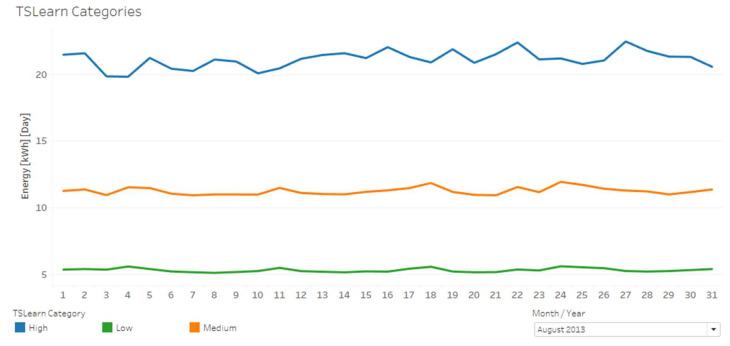
Average energy consumption for each day of August 2013 for each TSlearn category.

Identified clusters were denominated as high, medium, and low, referring to the electricity consumption of each group of clients. For example, Figure 5 presents a graph with the average energy consumption for each day of August 2013 for each TSlearn category.

For this clustering exercise, we run 50 experiments to guarantee repeatability since the assignment of centroids of each initial cluster was random at the beginning of each experiment.

Once each household had an assigned group, it was possible to make comparisons between the grouped categories and those found in this work, which is now called TSlearn categories. The results of the clustering task are presented later in Section 5.

##### Forecasting

As presented before in Section 2, forecasting is one of the main applications of AMI data analytics. A critical task of predictive analytics addressed in this work is forecasting.

For the forecasting application, this work uses the Nonlinear AutoRegressive eXogenous model (NARX) model, expressed as follows.

(2) $$\hat{y}\left(t+k\right)=f\left(\begin{array}{c} \hat{y}\left(t+k-1\right),\;\ldots ,\;\hat{y}\left(t+k-p\right),\\ \;x_{1}\left(t+k-1-d_{1}\right),\;\ldots ,x_{1}\left(t+k-d_{1}-q_{1}\right),\;\ldots ,x_{m}\left(t-d_{1}\right),\\ \;\ldots ,x_{m}\left(t-d_{m}-q_{m}+1\right) \end{array}\right)$$

where $\hat{y}$ is the variable to forecast, f is the estimation function, p is the autoregression order, q is the order of the exogenous inputs, and d represents a time delay in the exogenous inputs if it is necessary.

The estimation function f represents an estimator with a learning capacity. Some conventional estimators in machine learning are artificial neural networks, decision trees, and random forests [75].

On the one hand, artificial neural networks (ANN) are assemblies of single neurons that acquire knowledge from experience with historical data. The assemblies establish their learning parameters to predict a future value with the least possible error, which is similar to the learning process of a real neuronal system. On the other hand, decision trees are trees whose ramifications represent possible decisions made according to the characteristics of each record in the available data. In the end, after the decisions are taken based on the available characteristics, each record is assigned a final value or a class label depending on the machine learning application [78].

Due to their implementation simplicity, decision trees have evolved into more robust versions as random forests, which are ensembles of decision trees. A random forest operation proposes that several weak learners join to form a robust learner to increase predictive power and robustness for more extensive and more complicated datasets. These estimators have become widely used for predicting energy consumption [36,37].

For this case study, the estimation function (f) was a random forest. The variable to be forecasted ($\hat{y}$) was the energy consumption, and the environmental temperature was the exogenous input (x), given the high relation with the electricity consumption identified in the previous sections. The forecasting was made recurrently with hour granularity. The consumption of the previous 24 h defined the consumption value of the following hour, i.e., $\hat{y}\left(t+k\right)|\;k=1$. It defined the order of the auto—regressor p=24 and the exogenous inputs q=24.

The prediction was made recurrently for each hour of the day, each grouped category, and each meter. For this task, we used data for 2013. A total of 70% of the data was used for model training and 30% was used for testing. The library used for the forecasting algorithm was fireTS, which is an extension of Scikit-learn specialized in the prediction of time series [79].

The results of the clustering task are presented later in Section 5.

### 4.4. Visualization and Access

Data visualization and access support information analysis of the results presented as part of the information value chain presented in the NIST framework in Figure 1. For this work, part of the data used and generated corresponds to development stages like ETL and data warehouse building. In contrast, another part focuses on the presentation and visualization of results.

Since this study case was exploratory, the visualization of the data focused on the presentation of the results obtained for the two types of analytics addressed (descriptive and predictive analytics). We designed Tableau dashboards [80] to present results as detailed as the available data allowed. Its design aimed to provide global access for readers to all obtained results and not only for a specific profile, as previously mentioned in Section 2. For this case study, we developed five Tableau workbooks containing several dashboards to visualize the results.

The first two workbooks correspond to the results of descriptive analytics. They include a set of dashboards that display electricity consumption by customers with different time granularities (hour, day, month, and year) and a section to visualize the difference in consumption between workdays and holidays. Dashboards also include visualization of consumption and payments differentiated by tariff (Std or ToU).

The last three books condense the results of predictive analytics, which result from implementing the forecasting and clustering algorithms. The dashboards with the forecasting results allow seeing information disaggregated by customers and categories. The clustering dashboards present a comparison between the groups assigned to the TSlearn categories and the original grouped categories.

**Figure 6:**
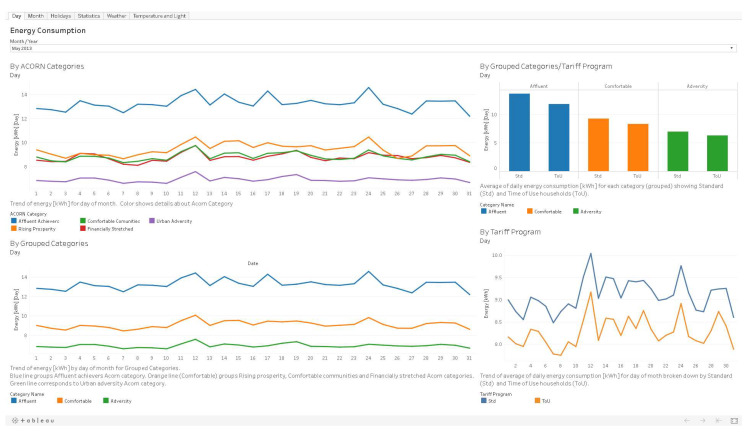
Descriptive analytics dashboard—Day of the month.In this way, information with different temporal granularities (hour, day, month), different household groups (acorn or grouped categories, Std or ToU households), analysis for specific events (holidays vs. business days), correlations with external variables (temperature, humidity, light hours), and comparisons with other types of results (groups assigned by the clustering algorithm and electricity consumption forecasting) were made available.

Figure 6 shows an example of the dashboards developed for result visualization corresponding to descriptive graphs with information per day each month. The upper left graph shows consumption per day for Acorn categories. The lower-left graph presents the same information for grouped categories. The right side shows the average consumption values by grouped categories and tariff program, while the lower-right graph presents consumption by day for each tariff program.

Throughout Section 5, we present some other graphs and dashboards designed for displaying results. The main contribution of the data visualization stage, which is transversal to tasks of descriptive or predictive analytics, is to facilitate the interpretation of results obtained when treating the information. Then end users can show knowledge that is not perceptible in the first instance and identify some trends or behaviors of interest quickly.

Below, we detail the results obtained in the implementation of the data analytics stages.

## 5. Data Analytics Results

This section presents the results of the data analytics tasks that we developed: descriptive analytics and predictive analytics that included clustering and forecasting algorithms.

### 5.1. Descriptive Analytics

**Figure 7:**
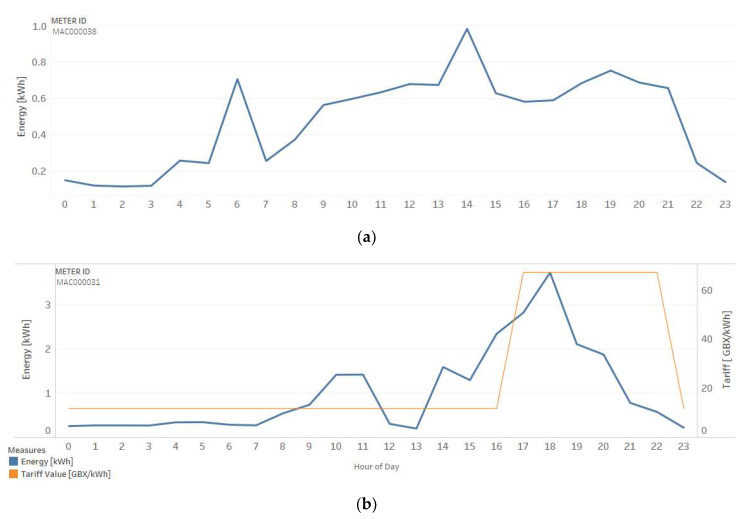
Household energy consumption: (**a**) metering with a standard tariff and (**b**) metering with a dynamic time of use.

Figure 7 shows the energy consumption per hour of two households (a) with a standard tariff (Std) and (b) with dynamic time of use (ToU), both on 13 April 2013. The blue line presents energy consumption (kWh), and the orange line corresponds to the tariff value (GBX/kWh), which is more expensive from 17 to 22 h.

While the customers with the standard tariff do not modify their consumption in these hours, customers with ToU tariff reduces the consumption sharply for the same period. Reduction of energy consumption through client awareness is one of the objectives of a demand response program.

Table 3 presents a summary of the consumption data of the grouped categories and their tariff program. The table also presents a column with the percentage difference between the consumption of users with Std and ToU tariffs.

Table 3 shows that customers with the ToU tariff program have a lower energy consumption, around 12%, than customers with the Std tariff. This difference between consumption shows the effectiveness of the demand response program implemented by the low carbon London program. It shows a change in the consumption patterns of the users who participated in the program through incentive/penalties in the tariff schemes, as described above. Furthermore, we can see how the most considerable percentage differences are in the affluent category, while the smallest percentages are in the adversity category. This behavior might be because users in the affluent group can economically perceive a more significant reduction in payment since they have a much higher average consumption than the adversity group. We can observe that, in the case of the adversity category, the indicator of maximum consumption per day shows an increase instead of a reduction in ToU users compared to Std users. However, the other indicators do show a decrease, although less than that observed for the affluent category. Since this is a maximum value, this increase may be due to isolated data that does not necessarily represent the generality of the adversity category. Instead, average values describe the regular behavior of users in each category.

**Figure 8:**
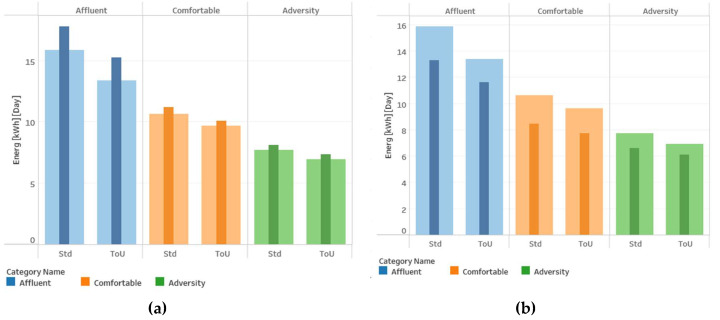
Energy consumption on holiday days (thin) versus workdays (thick): (**a**) year 2013 consumption, (**b**) August 2013 consumption.

There are also some other exciting findings in households’ consumption patterns. For example, a utility may find it useful to detail the consumption difference between a working day and a holiday, as observed in Figure 8.

**Figure 9:**
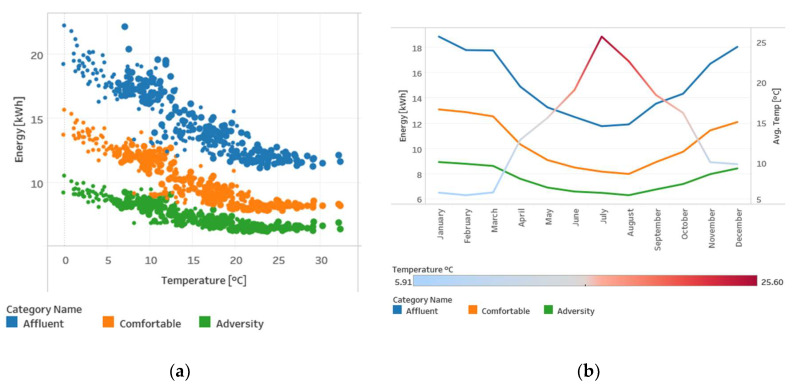
Energy consumption versus temperature: (**a**) scatter plot and (**b**) time-line plot.

Figure 8a shows that average daily consumption on holidays (thin bars) was higher than on workdays (thick bars) in 2013. However, if we focus only on August 2013, as shown in Figure 8b, the graph shows an inverse behavior. It may be due to households’ occupancy during months that are generally related to the vacation season and the temperature in the summer season, as observed in Figure 9.

**Figure 10:**
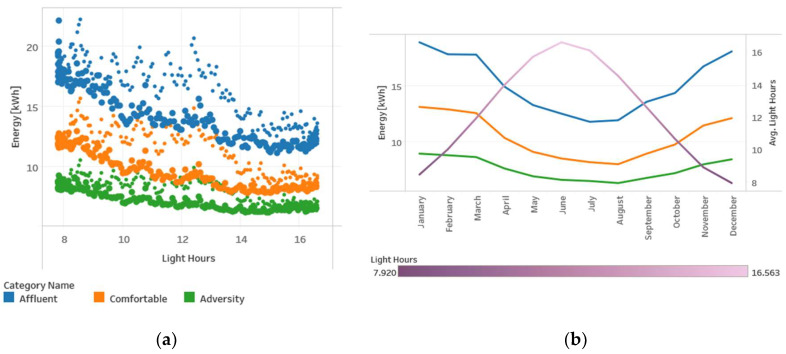
Energy consumption versus light hours per day: (**a**) scatter plot and (**b**) time-line plot.

Regarding weather variables, there were two of them with an evident relation with households’ consumption, temperature, and light hours per day. Figure 9a presents the relation between electricity consumption versus temperature per day in 2013. In addition to the inverse relationship identified between energy consumption and average temperature, Figure 9b shows that, along with the winter season, energy consumption reaches higher values. Something similar happens with the average light hours per day, as shown in Figure 10.

Figure 10 presents electricity consumption per day vs. light hours for each day of 2013. There is an inverse relationship between the light hours and energy consumption with a hysteresis noted in Figure 10a. Thin points represent consumptions measured during the first semester of the year, and thick points correspond to the last semester of the year for each of the three grouped categories.

### 5.2. Predictive Analytics—Clustering

For this task, we used the Silhouette score to measure the quality of the clustering exercise. This value varies from −1 to 1. A Silhouette score = 1 means that the clusters are well-differentiated or segmented and that elements inside each group are similar or closer to each other. The standard deviation of the Silhouette scores in the 50 experiments was 0.0049, which indicates algorithm repeatability.

The results of the clustering algorithm are presented below in Table 4.

**Figure 11:**
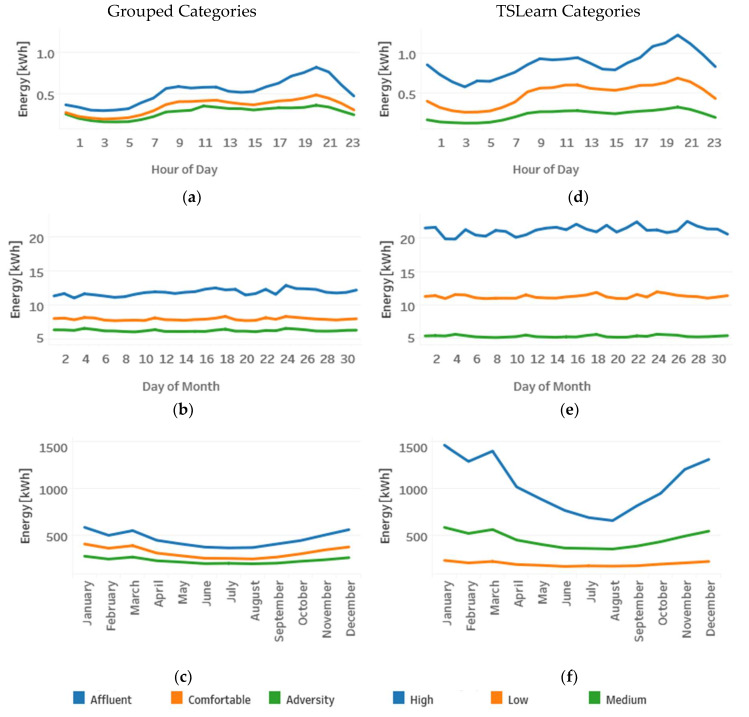
Energy consumption for grouped categories (**a**–**c**) vs. TSlearn categories (**d**–**f**).

Figure 11 presents the difference in the electricity consumption scales of the groups obtained with the TSlearn library when compared with the original grouped categories. The left side of the graph shows the average consumption per hour (a), day of the month (b), and month of the year (c) for the grouped categories. The right side of the graph correspondingly shows the average consumption per hour (d), day of the month (e), and month of the year (f) for the TSlearn categories. Due to the high Silhouette score obtained with the implemented clustering algorithm, it is possible to see a greater separation between TSlearn categories than the separation in the clusters of grouped categories.

**Figure 12:**
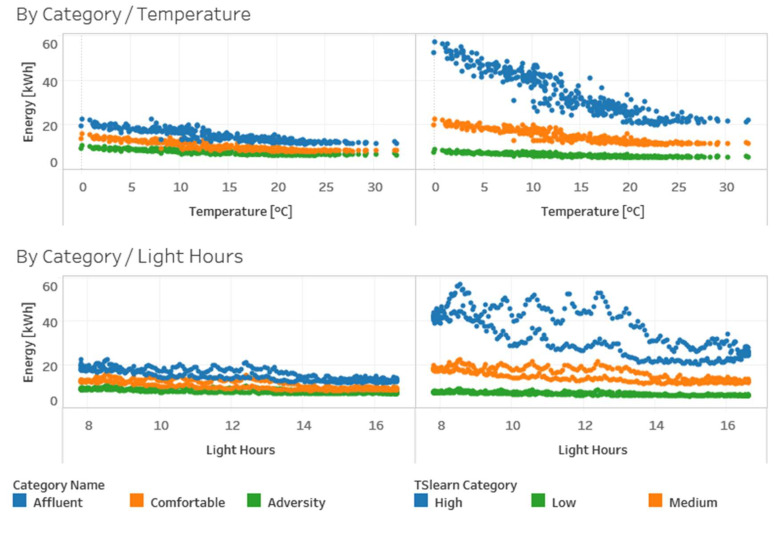
Energy consumption vs. temperature and light hours for grouped and TSlearn categories.

Figure 12 shows relationships between energy consumption and temperature or light hours for both grouped and TSlearn categories. Similarly, we can observe the difference between grouped categories and TSLearn categories.

As previously stated in Section 4.3, we intended the two segmentations (grouped and TSlearn) to be complementary and not exclusive. We implemented the TSLearn categories to verify if there is a correspondence between geo-demographic and consumption-based categories. However, the difference between the two categories shown in previous figures indicates that the assumption is not entirely accurate.

**Figure 13:**
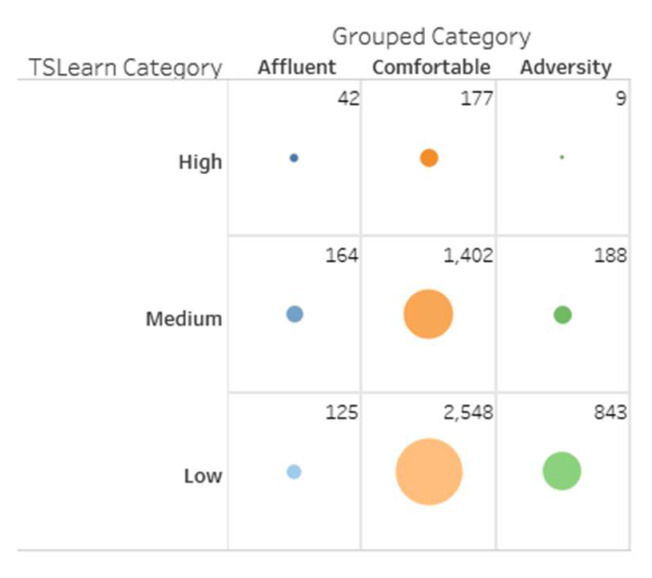
Energy consumption matrix for grouped categories vs. TSlearn categories.

Figure 13 presents a matrix that relates grouped categories with the TSlearn categories. Dots’ size indicates the number of users that simultaneously belong to a grouped category and a TSlearn category. The matrix shows that there is no total correspondence between the grouped categories and the TSlearn categories. For example, not all users with the affluent grouped category were in the high TSlearn category. Not all users from the comfortable category were in the medium category, and so on.

**Figure 14:**
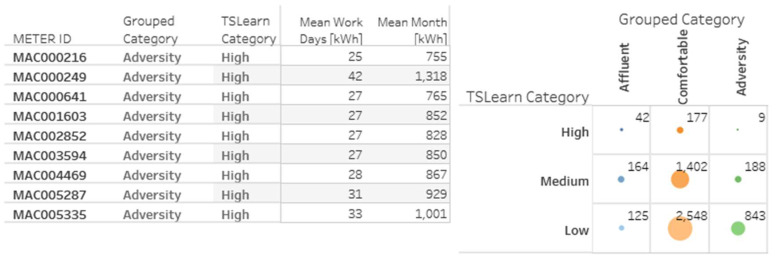
Households from the adversity category with high energy consumption.

However, as we said before, the idea behind proposing a new type of customer segmentation is not to dismiss their initial classification, but to find value in the information provided by both groupings. For instance, Figure 14 shows nine customers from the adversity category simultaneously allocated to the high TSlearn category. For a utility, it might be helpful to know what is happening with a customer who, belonging to an adversity category, registers high energy consumptions.

Other clients that may be subject to verification are those that, belonging to an affluent category, present low electricity consumption. These types of correlations can help to recognize, for example, commercial or industrial activities (with high demand for electricity) installed in residential sectors, imbalances in the nodes of the distribution network, or non-technical losses.

### 5.3. Predictive Analytics—Forecasting

Table 5 presents error measurements (R^2^ score, mean absolute error, root mean square error, and mean square error) obtained in the forecasting of energy consumption for each case including the average error measure obtained for all 5509 m and each category.

**Figure 15:**
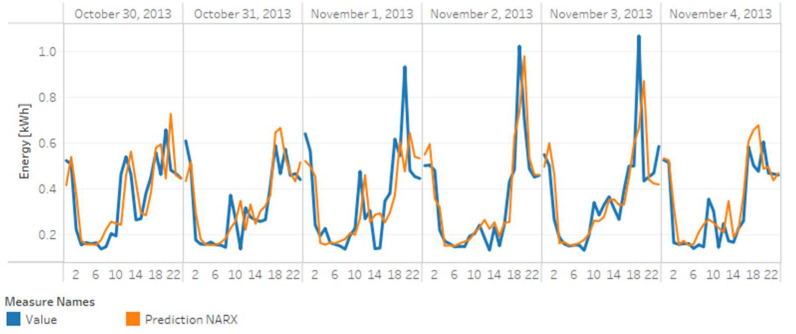
Electricity forecasting for meter MAC000002.

**Figure 16:**
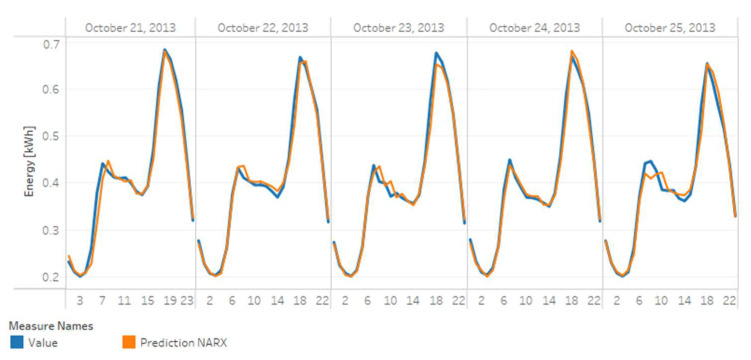
Electricity forecast for a comfortable category.

Figure 15 shows a time-series graph of the actual (blue line) and forecasted (orange line) hourly electricity consumption for a meter (MAC00002). Similarly, Figure 16 shows the forecast consumption for the comfortable group of grouped category.

In general, forecasting for the grouped categories has good performance indicators, unlike forecasting for individual meters. In that case, the performance was lower, considering that not all meters had the same amount of records available for the construction of their model.

## 6. Conclusions and Upcoming Developments

This paper presents the results of the application of big data and data analytics concepts in a case study with AMI data, taken from the smart energy meters in the city of London. The work developed shows the transformation of raw data into knowledge that allows rapid identification of trends, average and individual patterns, unusual events, and, in general, provides support for data-driven decisions aimed at the best planning/operation of the distribution system.

Data processing on a distributed platform allowed the consolidation of a data warehouse with more than 600 million records including aggregate information in different temporal granularities and categorical grouping. In addition, it includes external variables that are highly related to the patterns of consumption for about 5,500 households.

Descriptive analysis showed how households that participated in a dynamic time of use (ToU) tariff program had lower electricity consumption, which indicates the positive impact that the implementation of a demand response program can have when supported by the installation of Advanced Metering Infrastructure (AMI).

There is a high relation between variables like temperature and light hours with users’ electricity consumption. In addition, it is important to notice how these changes influence specific consumption habits. For example, the consumption between workdays is, in general, lower than during holidays. However, this behavior is different in the summer months, which may be related to seasonal changes in temperature.

Given that the number of elements to be grouped was relatively small (5509 households) and we knew the number of clusters to group users (we aimed to get three groups), K-means was a fit candidate as a clustering algorithm. However, the potential application of this clustering task can grow up to hundreds of millions of users [13,14]. In those cases, there are other algorithms like batch k-means, spectral clustering, or Variational Bayesian Gaussian Mixture (VBGM), according to each case requirement [76].

The application of clustering techniques (TSlearn categories, consumption-based) allowed proposing a new household segmentation different from the one assigned initially (Grouped Categories, geo-demographic based). When compared, these two types of categories allowed identification of users with atypical patterns. It might represent industrial or commercial activities in residential sectors, technical or non-technical losses or even help planning better pricing schemes for demand response programs.

The computational resources used for data processing (a distributed data processing cluster) facilitated the implementation of the case study from the role of a big data framework provider. For example, this includes consolidation into a distributed framework of a data warehouse. This consolidation, which involved managing millions of records, was a simple but time-consuming task. Future work might include a test benchmark to evaluate the performance of this type of application, depending on the computational infrastructure detected.

The forecasting task presents one of the main challenges in the analysis of AMI data. In this case study, the implemented algorithm presented significantly low error indicators, even in the case of individual meters. The algorithms implemented in this case study are not mandatory. Nevertheless, the results show the potential of combining an autoregressive algorithm (NARX) with an ensemble of learning entities (Random Forest) and libraries specialized in time series data (fireTS) as part of the data life cycle for this type of application.

For future work, we propose a more sophisticated development of the stages of visualization and access. As mentioned in Section 2, a more ambitious implementation of a data analytics application for a utility may require result visualization for people with different roles within the company. Thus, visualization and access must explicitly support the work of each role. Some works from Section 2 mention several approaches to these issues [59,61].

Furthermore, the inclusion of a sentimental analysis of information might be useful. That is the correlation of electricity consumption with events that can show trends in social networks: international impact news, world-order events such as Olympic Games, FIFA World Cups, and others.

## Figures and Tables

**Table 1:** Works on Advanced Metering Infrastructure (AMI) data applications.

AMI Application	Related Works
AMI data processing platforms.	[18,19,20,22]
Linking emerging technologies in AMI data processing.	[54,55]
Identification of consumption profiles from AMI data.	[23,24,26,28]
AMI data for loss reduction.	[51,52]
AMI data for demand response programs.	[4,48,49]
Load forecasting using AMI data.	[27,38,39,40,41,42,43,44,45,46]
Load profile disaggregation (identification of devices and household appliances connected to the network)	[29,30,31]

**Table 2:** Initial data frames generated from data sources.

Data Frame Name	Description	Number of Records
uk_hd	UK bank holidays	25
acorn_cats	Acorn categories and population percentage	6
acorn_groups	Acorn groups and their categories	18
information_households	Information about household’s meters	5517
weather_daily_darksky	Weather information per day from 2011 to 2014	882
weather_hourly_darksky	Weather information per hour from 2011 to 2014	21,165
tariff_ts	Tariff timeline in a 30-min interval for ToU users through 2013	17,518
hh_ts	Time series - household’s consumption per half hour	165,809,909

**Table 3:** Data summary of average energy consumption per grouped categories and tariff program for 2013.

	Affluent			Comfortable			Adversity		
	Std	ToU	Percent Reduction	Std	ToU	Percent Reduction	Std	ToU	Percent Reduction
Average consumption per day (kWh)	15.92	13.42	15.70%	10.63	9.67	9.03%	7.72	6.95	9.97%
Maximum consumption per day (kWh)	26.38	20.29	23.09%	16.96	15.07	11.14%	12.42	15.9	−28.02%
Minimum consumption per day (kWh)	6.02	3.295	45.27%	7.36	6.11	16.98%	5.53	2.50	54.79%
Average consumption per workday (kWh)	15.55	13.36	14.08%	10.44	9.53	8.72%	7.49	6.82	8.95%
Average consumption on Sundays (kWh)	16.69	14.46	13.36%	11.25	10.24	8.98%	8.1	7.58	6.42%
Average consumption on holidays (kWh)	17.71	15.77	10.95%	11.17	10.08	9.76%	7.99	7.27	9.01%
Average conception per month (kWh)	484.52	415.48	14.25%	324.22	294.47	9.18%	232.68	212.61	8.63%
Maximum consumption per hour (kWh)	1.79	1.62	9.50%	1.35	1.25	7.41%	1.05	0.93	11.43%
Time of day of maximum consumption	18	17		17	17		17	17	
Number of Users	255	76		3253	874		883	157	

**Table 4:** Result summary of the k-means clustering algorithm.

Indicator	Value
Number of clusters	3
Number of features	24 ^1^
Objects in dataset	5509
Number of experiments	50
Average silhouette score	0.7815
σ Silhouette score	0.0049
Execution time	5 min 27 s

^1^ Corresponding to each hour of the day.

**Table 5:** Result summary for electricity forecasting algorithm.

Category	R^2^	MAE (kWh)	RMSE (kWh)	MSE (kWh^2^)
Individual Meters	0.6585	0.1161	0.1240	0.0154
Affluent	0.9741	0.0301	0.0435	0.0019
Comfortable	0.9835	0.0145	0.0211	0.0004
Adversity	0.9732	0.0118	0.0160	0.0003
